# A duty to act

**DOI:** 10.1128/aac.01104-26

**Published:** 2026-09-11

**Authors:** Emily Sickbert-Bennett, Philip J. Rosenthal, Cesar A. Arias, Gonzalo M. Bearman, Paul E. Sax, Graeme Forrest, David P. Calfee, Romney M. Humphries, Stanley Maloy, Ravi Jhaveri, Ira Blader, Ashley Shade, Roger Bedimo, Cynthia L. Sears, Paul Katz, Barbara Resnick

**Affiliations:** 1 American Journal of Infection Control; 2 American Journal of Tropical Medicine and Hygiene; 3 Antimicrobial Agents and Chemotherapy; 4 Antimicrobial Stewardship & Healthcare Epidemiology; 5 Clinical Infectious Diseases; 6 Clinical Microbiology Reviews; 7 Infection Control & Hospital Epidemiology; 8 Journal of Clinical Microbiology; 9 Journal of Microbiology & Biology Education; 10 Journal of the Pediatric Infectious Diseases Society; 11 mSphere; 12 mSystems; 13 Open Forum Infectious Diseases; 14 The Journal of Infectious Diseases; 15 Journal of the American Medical Directors Association

**Keywords:** healthcare, public health, research vaccines, misinformation

## EDITORIAL

Throughout our history, the healthcare and scientific communities have united in the face of public health crises to save lives. During natural disasters like hurricanes or wildfires, infectious disease outbreaks and pandemics, and longstanding threats like tobacco use, robust and unified responses from healthcare professionals and scientists have delivered information, innovation, and care that have empowered millions of Americans to lead longer, healthier, and more fulfilling lives (1, 2).

Taken together, the current spread of misinformation by federal health agencies and the dismantling of much of our public health and medical research infrastructure and healthcare safety net constitute another crisis that has already claimed American lives and is poised to do even greater damage to our nation’s health. Once again, healthcare professionals and scientists have a duty to act. Our patients and communities are counting on us.

The impacts of the current administration’s actions rise above typical partisan disagreements to pose a major threat to the health of the American people. Consider the following examples:

- Access to healthcare: The Congressional Budget Office estimates that about 10 million people will lose health insurance as a result of the so-called Big Beautiful Bill. That will result in more people getting delayed diagnoses and care, leading to poorer outcomes, higher costs, and more emergency department utilization (3).
- Healthcare workforce: To further increase barriers to care, the administration is cutting clinician reimbursement, downgrading the essential profession of nursing to restrict access to student loan forgiveness programs, and limiting the ability of health professionals from other countries to work in the United States by tampering with our visa systems. All of these policies strike at the heart of our healthcare workforce, curtailing recruitment and retention of highly trained professionals who were already suffering workforce shortages and burnout (4–6).
- Research: America’s scientific research engine has long been a world leader, inspiring generations of researchers to deliver new tools to prevent, detect, and treat diseases, lengthening the human lifespan and improving our quality of life. Scientific breakthroughs take years and require predictability of funding. This administration introduced chaos into our grantmaking processes, arbitrarily freezing grants, proposing massive funding cuts, halting international scientific collaborations, and injecting politics into longstanding grant review processes. This will slow the pace of lifesaving scientific advances, discourage a generation from pursuing research careers in the United States, cause our nation to fall behind globally, and leave Americans less prepared to face the next health threat (7).
- Public health: The exodus of experts from the Centers for Disease Control and Prevention (CDC) and other Health and Human Services (HHS) agencies coupled with the loss of funding for state and local health departments leaves Americans profoundly less safe, less prepared for emergencies, and less capable of tackling public health problems like obesity, tobacco use, substance abuse, and outbreaks of infectious diseases. The administration’s cuts will delay and limit our ability to identify and track new pathogens, which will allow more people to get sick and slow our ability to offer testing, prevention strategies, and optimal care (8).
- Vaccines: The purposeful erosion of vaccine confidence, access, and uptake is already having devastating effects on our population, as evidenced by the explosion of measles cases in the United States. The administration’s dismissal of CDC’s independent expert vaccine advisors and replacement with politically influenced science deniers have created significant confusion and chaos among patients, families, and clinicians; restricted access to lifesaving vaccines; and significantly damaged the credibility of what was once the premier public health agency in the world. While recent legal actions have stayed most of the new appointments and votes of the CDC Advisory Committee on Immunization Practices and the HHS changes to the pediatric immunization schedule, many legal issues are still pending, and it will take a significant and concerted effort to rebuild U.S. vaccine infrastructure and help the public access and discern credible vaccine information.

It is important to recognize that while the assault on science and health impact us all, the most vulnerable—low-income individuals; people of color; those living in rural communities; immigrants; lesbian, gay, bisexual, transgender, and queer (LGBTQ) people; older adults; babies; and the immunocompromised and others with significant medical needs—are bearing the brunt of the consequences. As healthcare professionals and scientists, we have always taken care of those in greatest need, and the need has never been greater than it is right now.

While it is easy to despair at the breadth and barrage of attacks on our health, we have an ethical obligation to our patients and communities to resist these changes. We must do more than simply aim to protect the status quo. Let’s face it—deep dissatisfaction with the status quo is, in part, what led us to the current moment. We know that our longstanding systems were steeped in inequities and failing too many people. We must seize this opportunity to create something far better: a low barrier, person-centered healthcare system that serves everyone, a stronger public health infrastructure that meets the needs of all communities, and innovative medical research that prioritizes patients. But how?

As healthcare professionals and scientists, our training and lived experiences provide us with a wealth of knowledge that we can use to stand up for science and improve our nation’s health in a systemic way. In addition, our professional roles make us trusted voices in our communities. Banding together, we can elevate our voices further for the betterment of all. What can that look like?

- Educate: We have many opportunities to educate colleagues, family members, patients, and friends, by listening to their concerns and responding with factual information and without judgment. We can clearly explain how different policies are impacting us and those around us. Working with our professional societies, we can use traditional and nontraditional media to get our messages out to wider audiences. A better educated populace can more effectively sway their elected officials and make more informed decisions in future elections.
- Advocate: We can also urge our lawmakers to base policies on the best available scientific evidence and provide adequate funding for healthcare, public health, and medical research. Our professional societies provide an array of tools to support advocacy, including trainings, talking points, online mechanisms to contact Congress, advocacy days, and more. It is critical that senators and representatives hear from their constituents and face pressure from their states and districts to stand up to the administration and advance policies that align with their constituents’ best interests.
- Run: Fundamentally, if we want better policies, we need better policymakers. Who better than healthcare professionals and scientists to drive a policy agenda steeped in science and geared toward improving the health of all? The 314 Action PAC provides support to science, technology, engineering, and mathematics (STEM) professionals who run for office at the state or national level, recognizing that our voices are essential in the halls of Congress and our state legislatures.

Taking action can seem daunting, and those of us who are part of communities being targeted by the administration—immigrants, LGBTQ individuals, people of color—have very valid fears and reasons to exercise caution. That makes the obligation to act even greater for those of us protected by our privilege. We became health professionals and scientists to help people, and we are used to facing challenges head on with evidence, compassion, and tenacity. Those are the very qualities our nation needs now.

## EDITOR'S NOTE

**Ed. Note:** This article has been co-published with permission in *Clinical Infectious Diseases*, *The Journal of Infectious Diseases*, *Open Forum Infectious Diseases*, *Journal of the Pediatric Infectious Diseases Society*, *Antimicrobial Agents and Chemotherapy*, *Clinical Microbiology Reviews*, *Journal of Clinical Microbiology*, *Journal of Microbiology & Biology Education*, *mSphere*, *mSystems*, *Antimicrobial Stewardship & Healthcare Epidemiology*, *Infection Control & Hospital Epidemiology*, *Journal of the American Medical Directors Association*, *American Journal of Infection Control*, and *The American Journal of Tropical Medicine and Hygiene*. The articles are identical except for minor stylistic and spelling differences in keeping with each journal’s style. Any citation can be used when citing this article.
